# Wild vascular plants gathered for consumption in the Polish countryside: a review

**DOI:** 10.1186/1746-4269-3-17

**Published:** 2007-04-15

**Authors:** Łukasz Łuczaj, Wojciech M Szymański

**Affiliations:** 1 High School of Humanities and Economics in Łódź, Department of Humanities, ul. Rewolucji 1905 r. nr 64, 90-222 Łódź, Poland; 2 ul. Witosa 6/18, 28-400 Pińczów, Poland

## Abstract

**Background:**

This paper is an ethnobotanical review of wild edible plants gathered for consumption from the end of the 18^th ^century to the present day, within the present borders of Poland.

**Methods:**

42 ethnographic and botanical sources documenting the culinary use of wild plants were analyzed.

**Results:**

The use of 112 species (3.7% of the flora) has been recorded. Only half of them have been used since the 1960s. Three species: *Cirsium rivulare*, *Euphorbia peplus *and *Scirpus sylvaticus *have never before been reported as edible by ethnobotanical literature.

The list of wild edible plants which are still commonly gathered includes only two green vegetables (*Rumex acetosa *leaves for soups and *Oxalis acetosella *as children's snack), 15 folk species of fruits and seeds (*Crataegus *spp., *Corylus avellana*, *Fagus sylvatica*, *Fragaria vesca*, *Malus domestica*, *Prunus spinosa*, *Pyrus *spp., *Rosa canina*, *Rubus idaeus*, *Rubus *sect. *Rubus, Sambucus nigra, Vaccinium myrtillus, V. oxycoccos, V. uliginosum, V. vitis-idaea*) and four taxa used for seasoning or as preservatives (*Armoracia rusticana *root and leaves, *Carum carvi *seeds, *Juniperus communis *pseudo-fruits and *Quercus *spp. leaves). The use of other species is either forgotten or very rare.

In the past, several species were used for food in times of scarcity, most commonly *Chenopodium album*, *Urtica dioica*, *U. urens*, *Elymus repens*, *Oxalis acetosella *and *Cirsium *spp., but now the use of wild plants is mainly restricted to raw consumption or making juices, jams, wines and other preserves. The history of the gradual disappearance of the original *barszcz*, *Heracleum sphondylium *soup, from Polish cuisine has been researched in detail and two, previously unpublished, instances of its use in the 20^th ^century have been found in the Carpathians. An increase in the culinary use of some wild plants due to media publications can be observed.

**Conclusion:**

Poland can be characterized as a country where the traditions of culinary use of wild plants became impoverished very early, compared to some parts of southern Europe. The present use of wild plants, even among the oldest generation, has been almost entirely restricted to fruits.

## Background

Wild plants, even after the advent of agriculture, constituted an important part of the human diet, especially in poor rural communities. Wars and times of famine were periods when the knowledge of such plants was especially important for communities [1]. Although there is huge data on the medicinal and culinary use of plants in Europe, the available material is usually dispersed in small ethnographic papers published in native languages. There is not an up-to-date exhaustive study on all Europe's edible plants, comparable with Moerman's *Native American Ethnobotany *[2], except for a popular guide by Couplan [3]. Although attempts to compile worldwide lists of wild edible plants exist [4-7], they are far from exhaustive.

Recent regional studies, especially from the Mediterranean part of Europe, like some regions of Spain [8-11], Italy [12-15] and Cyprus [16], as well as the comparison of several regions of Spain, Italy and Greece [17], have shown that the continent has a rich and varied culture associated with the culinary use of wild plants. Reviews on a national scale, concerning the ethnobotany of wild food, have also been published, e.g. reviews of wild edible plants used in Spain [18] and Bosnia-Herzegovina [19], a monograph of edible green vegetables of Italy and a list of potentially edible plants of Slovenia [20].

This paper is an attempt to present a checklist of food plants collected from the wild in another European country – Poland. The authors hope that this review will be a building block in a monograph of wild edible plants of Europe, encompassing the traditions of all European nations. Poland lies in the centre of Europe, and thus shares a large proportion of flora with its neighbouring countries, so the knowledge of traditional use of its plants may be very valuable. However, due to language difficulties, most Polish ethnobotanical literature is not known outside the country, except for the brilliant work of Maurizio [1], published also in German [21] and in French [22].

**Figure 1:**
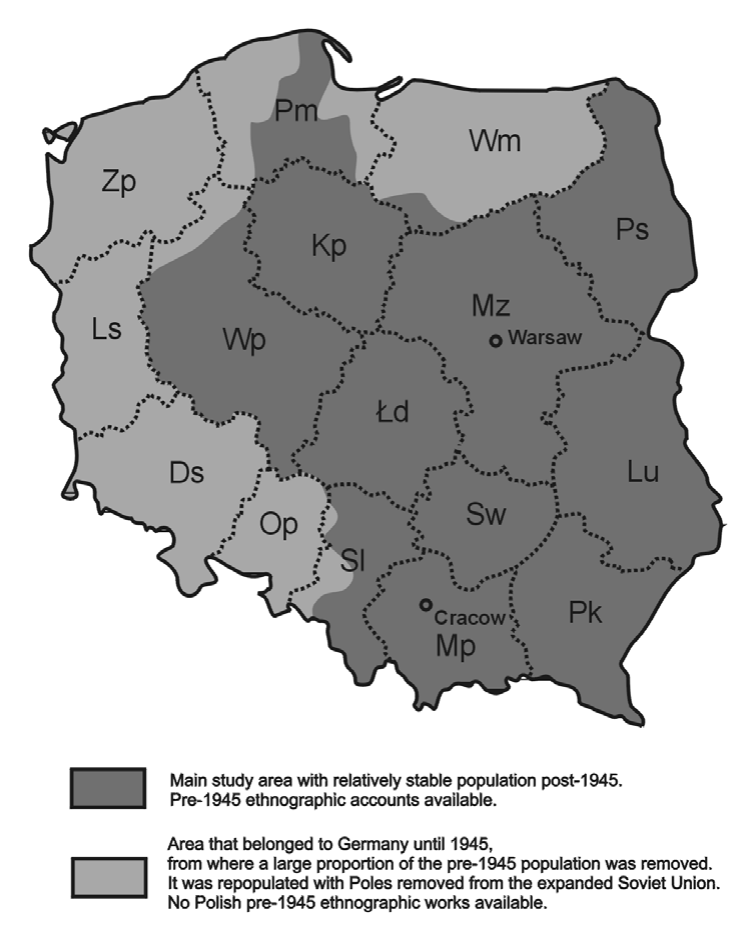
Study area with the present administrative division of Poland into 16 *województwo *regions. Abbreviations: Ds – Dolnośląskie, Kp – Kujawsko-Pomorskie, Ls – Lubuskie, Lu – Lubelskie, Łd – Łódzkie, Mp – Małopolskie, Mz – Mazowieckie, Op – Opolskie, Pk – Podkarpackie, Pm – Pomorskie, Ps – Podlaskie, Sl – Śląskie, Sw – Świętokrzyskie, Wm – Warmińsko-Mazurskie, Wp – Wielkopolskie, Zp – Zachodniopomorskie.

Poland is a middle-sized European country, with an area of 312 thousand km^2^, slightly smaller than Germany and larger than Italy (Fig. 1). Although some climatic variation occurs, it is a country with a relatively uniform cold temperate climate and a large proportion of lowland areas. Natural potential vegetation is predominantly deciduous woodland (with the dominance of *Quercus robur*, *Carpinus betulus*, and, in the south and west, *Fagus sylvatica*) with some coniferous woodland (mainly *Pinus sylvestris *and *Picea abies*) in poorer soils, however the dominant type of present forest vegetation is *Pinus sylvestris *plantations. The vascular flora of Poland contains approximately 3000 species, including the better-established aliens [23].

Polish borders have shifted a few times. The last such shift occurred after World War II, when, as a result of the decision of world's superpowers, the country's borders were shifted some 200 km westwards (Fig. 1). Because of this, accounts of the use of some plants in Polish pre-1939 ethnobotanical literature often come from areas now situated near the eastern Polish border – in Lithuania, Belarus or Ukraine – inhabited even just before World War II largely by non-Polish populations. The aim of this study is to present a full list of all vascular plants which have been eaten within the present area of Poland since the end of the 18^th ^century. Thus, for the sake of clarity, we have excluded information on the consumption of plants within the present borders of Ukraine, Lithuania, Belarus and Russia, often occurring in older Polish ethnobotanical literature due to historical and geographical affiliations. The authors did not search German ethnographic literature which might have quoted the use of edible plants in the areas of western and southern Poland (Silesia, Pomerania) which used to be part of Germany before World War II, although we have included post-World War II references from these areas.

A very important factor shaping people's interest in wild plants as food are times of famine or food scarcity. There is little evidence of widespread famine in Poland in the last 300 years, like that ones which occurred in China, Ukraine or Ireland, although a large proportion of the rural population in the 19^th ^century were undernourished [24]. Obviously some years were better than others, for instance Maurizio mentions years 1844–1897, as particularly bad for agriculture, and abounding in food shortages [1]. The problem increased throughout the 19^th ^century with strong population growth, but then it was alleviated at the turn of the 19^th ^and 20^th ^century by mass emigration to North and South America. Another period of strong under-nourishment was World War I, particularly its last two years, when Germany and Austria, two of the three countries occupying Poland, organized wild food collection points [1]. Characteristic feature of the Polish countryside were regular shortages of food in spring, when winter stores of grains and potatoes were running out. There is even a regularly used word in the Polish language, *przednówek*, literally 'before the new crops', which refers to the period of spring, which was commonly associated with hunger. Poland was for centuries an exporter of grain to the west of Europe, so with its large area under cultivation and large proportion of flat, easily cultivated land it was not as prone to hunger as more mountainous countries. On the other hand its southern part has always been very densely populated, so that many large families have had to live off a plot of land smaller than one hectare. Hence a popular expression was coined, *nędza galicyjska*, literally Galician poverty, referring to the south of Poland, which constituted the province of Austro-Hungarian empire named Galicja (Galizien in German) [24].

Eating wild products is becoming fashionable in our post-modern society. Articles on the use of wild plants appear in popular magazines, which can influence their use. However, this is not a new phenomenon, and, since the existence of print, herbals and periodicals have published such information. Thus we can never be sure whether the recorded use is a local ancient tradition or a custom created by printed materials or medieval herbalists. Hence the approach of the authors towards the literature was very critical and mainly ethnographic literature was taken into account, or those popular articles and books, which reported firsthand the traditional use of certain plants.

Polish ethnobotanical literature on wild foods is not very rich, but has quite a long history and very rich traditions in mapping ethnographic phenomena. The most important source from the turn of the 18^th ^and 19^th ^century is the herbal of Krzysztof Kluk [25], a priest in Ciechanowiec (NE Poland). Among medicinal properties he also included the edibility of a species. As he often quotes information from foreign sources, in our checklist we only included the species which were clearly used in Poland, which can be guessed from expressions like "simple people gather it", "in our country" and the like. Another work of great importance is the monograph of wild edible plants by Adam Maurizio [1,22,23]. His monograph attempts to trace the gathering of wild plants across the world, but focuses mainly on Europe and Siberia, containing many references to Poland. Other important papers in this field include Rostafiński's work on the history of the use of *Heracleum *[26], Moszyński's monograph on Slavic folk culture which contains a detailed chapter on eating wild plants with many personal observations of the author [27], and Henslowa's monograph of the consumption of the genera *Chenopodium*, *Rumex*, *Sambucus*, *Urtica *and *Atriplex *[28].

The first list of edible plants of Poland was published by Mowszowicz [29]. He earlier published a similar paper on spices [30]. However this author did not include detailed references about the origin of his information and he included all potentially edible plants, especially plants consumed in other Slavic countries. Thus, unfortunately, this work could not be taken into account. A very important step in getting some deeper insight into the consumption of wild plants was the series of volumes of *The Ethnographic Atlas of Poland*, whose questionnaire included some questions concerning wild edible plants. A large proportion of data was collected in 1948–49 and 1964–69 [31], and generally gave the impression of a nation which had already lost, to a large extent, the tradition of consuming wild food other than mushrooms and wild fruits (the former especially, are still a living part of the nation's culinary culture). Maps on the use of most important wild plants were published in volumes 5 and 6 of the atlas [32,33]. Volume 7 was also going to contain maps of the use of some plants, but it has never been printed and is stored as a publicly available manuscript in the archive of the Polish Ethnographic Atlas at the University of Silesia in Cieszyn [34]. Fortunately its content was briefly discussed by its author, the late Janusz Bohdanowicz, in the commentaries to the Atlas, which contain his review of the main wild plants gathered in the Polish countryside [31]. Some of the data collected for the atlas were also summarized by Jędrusik [35].

Also the work of Łuczaj [36] is worth mentioning. He published a popular but very detailed guide containing a full list of potentially edible plants in the Polish flora, plus some of his own ethnographic observations. Although this piece of work is not fully referenced and contains data on the use of these plants in other countries and continents, a few detailed original descriptions of the use of certain plants, in certain areas of Poland, can be found.

Other papers quoted in our review are usually regional ethnographic monographs of material culture or traditional food in particular, which include references to wild edible plants in their chapters about local food. The first to mention among them is a series of 19^th ^century volumes on the ethnography of Poland written by Oskar Kolberg, who sometimes gave short descriptions of edible plants [37-41], then other studies followed, practically in all regions of the country [42-64]. The region most intensely studied by ethnographers was the Western Carpathians (particularly the Tatra Mountains, Podhale, Spisz and Orawa), where traditional culture was preserved including various food habits [41-51,63,64]. Altogether in this review, apart from the publications of the Polish Ethnographic Atlas, we used 12 general ethnographic papers on local rural culture, 6 local food monographs, 8 papers focused on wild food gathering practices in the countryside and 3 ethnomedical papers including Paluch's monograph [65] of plants used in Polish folk medicine (Tab. 1).

**Table 1:** Characteristics of literature sources used to make the list of species in the Appendix.

Reference No.	Author's name	Main topic	Research area	Region code	No. of species used
[1]	Maurizio (1926)	plant food	Eurasia and N America		15
[25]	Kluk (1786)	plant encyclopaedia	NE Poland		12
[26]	Rostafiński (1916)	food history	Whole country		1
[27]	Moszyński (1929)	material culture	All Slavic countries		19
[28]	Henslowa (1962)	selected edible taxa	Whole country		4
[30]	Mowszowicz (1969)	spices	Whole country		2
[31]	Bohdanowicz (1996)	ethnographic (food)	Whole country		26
[32]	Polish Ethnographic Atlas, Volume 5 (1974)	ethnobotanical maps (380 villages)	Whole country		5
[33]	Polish Ethnographic Atlas, Volume 6 (1981)	ethnobotanical maps (380 villages)	Whole country		9
[34]	Bohdanowicz, manuscript	ethnobotanical maps (380 villages)	Whole country		16
[35]	Jędrusik (2004)	gathering of wild plants (82 villages)	Whole country		11
[36]	Łuczaj (2004)	edible plant guide	Whole country		8
[37]	Kolberg (1888)	ethnographic (general)	Whole region	Mz, Wm	2
[38]	Kolberg (1888)	ethnographic (general)	Whole region	Mz, Ps	1
[39]	Kolberg (1890)	ethnographic (general)	Whole region	Lu	1
[40]	Kolberg (1890)	ethnographic (general)	Whole region	Lu	6
[41]	Kolberg (1968)	ethnographic (general)	Whole region	Mp	1
[42]	Eljasz-Radzikowski (1897)	ethnographic (general)	Tatra Mountains	Mp	5
[43]	Sarna (1898)	ethnographic (general)	Krosno	Pk	1
[44]	Sulisz (1906)	ethnographic (general)	Ropczyce	Pk	1
[45]	Jostowa (1954)	ethnographic (food)	Podhale	Mp	17
[46]	Wacławski (1965)	ethnographic (food)	Gorlice	Mp	13
[47]	Piekło (1971)	ethnograhic (foraging)	Brenna near Cieszyn	Sl	5
[48]	Kopacz (1976)	ethnograhic (foraging)	Jurgów, Spisz	Mp	7
[49]	Doliński (1982)	ethnograhic (foraging)	Łapsze Niżne, Spisz	Mp	14
[50]	Janicka-Krzywda (2004)	ethnographic (food)	Mount Babia Góra	Mp	11
[51]	Udziela (1994)	ethnographic (general)	Biecz	Mp	1
[52]	Chętnik (1936)	ethnographic (food)	Kurpie	Mz	16
[53]	Dekowski (1973)	ethnobotanical (foraging)	Kozienice forest	Mz	30
[54]	Dekowski (1968)	ethnobotanical (food)	Łowicz	Łd	18
[55]	Libera, Paluch (1993)	ethnomedical	Kolbuszowa	Pk	8
[56]	Szot-Radziszewska (2005)	ethnomedical	Whole region	Sw	5
[57]	Dydowiczowa (1964)	ethnographic (foraging)	Whole region	Wp	6
[58]	Skłodowska-Antoniewicz (1965)	ethnographic (foraging)	Złotów	Wp	2
[59]	Szromba-Rysowa (1966)	ethnobotanical (foraging)	Siołkowice	Op	13
[60]	Łęga (1960)	ethnographic (general)	Świecie	Kp	5
[61]	Łęga (1961)	ethnographic (general)	Ziemia Chełmińska	Kp	9
[62]	Malicki (1971)	ethnographic (foraging)	Pomerania	Pm, Kp	16
[63]	Jostowa (1954)	ethnographic (food)	Tatra Mountains	Mp	5
[64]	Wysłouchowa (1896)	ethnographic (general)	Wisła near Cieszyn	Sl	2
[65]	Paluch (1984)	ethnomedical	Whole country		17
[85]	Hryniewiecki (1952)	botanical monograph of fruits and seeds	Whole country		2

It must be stressed that the presented list of plants does not include records earlier than Kluk's work from 1786, e.g. studies of medieval cuisine and studies of archaeological remains from prehistoric times dealt with in other papers [66-69]. This review concentrates on food, including soups, jams, juices, sap and wines, but does not include herbal infusions or decoctions, difficult to deal with as they are most often drunk for medicinal purposes, with the exception of *Tilia *flower infusion and roasted acorn infusion which used to be in common everyday use throughout the country.

## Methods

In the review we analyzed a possible full list of 42 ethnographic and botanical publications including unpublished master's theses manuscripts, documenting the culinary use of wild plants within the present area of Poland, since the publication of Kluk's herbal at the end of the 18^th ^century.

The majority of these papers contain Latin names of plants, except for three smaller ethnographic papers [43,46,51]. No herbarium specimens are available to confirm the proper identification in the cited works, however we tried to, at least partly, verify the identification using the recently published atlas of the distribution of vascular plants in Poland [70] and generally available floras and plant guides. Every time there was a discrepancy between the identification in the literature and our view on the taxonomic status of the recorded used plant, we included a note in the list of plants. In a few cases we ascribed a genus (or a folk species encompassing it) to a particular species, when that was the only species occurring in the area, e.g. in one case we changed the identification from *macierzanka *(Polish for *Thymus*) [46] to *Thymus pulegioides*, as this is the only species from the genus occurring in the Gorlice area. On the other hand we applied extreme caution looking at accounts in which we spotted an obvious botanical mistake, e.g. suggesting that Latin names of plants were added by automatically looking up names in a plant guide without deeper knowledge of botany, e.g. mistaking *Origanum *and *Chenopodium*, because of the similarity in Polish names (*lebiodka *and *lebioda*, respectively) [31,44].

In the case when a commonly used plant was identified to a genus level, which comprises two or three very common species, not distinguished by folk taxonomy, we assumed that they were all used. For instance we assumed that both *Quercus robur & Q. petraea *were utilized, but we omitted *Q. pubescens*, which is extremely rare. In the case of the *Rubus *subgenus *Rubus *(i.e. *Rubus *sect. *Rubus *plus *R. caesius*), which constitutes one folk species, but comprises many botanical species, we listed four species, commonest in Poland, which we personally witnessed being collected.

**Figure 2:**
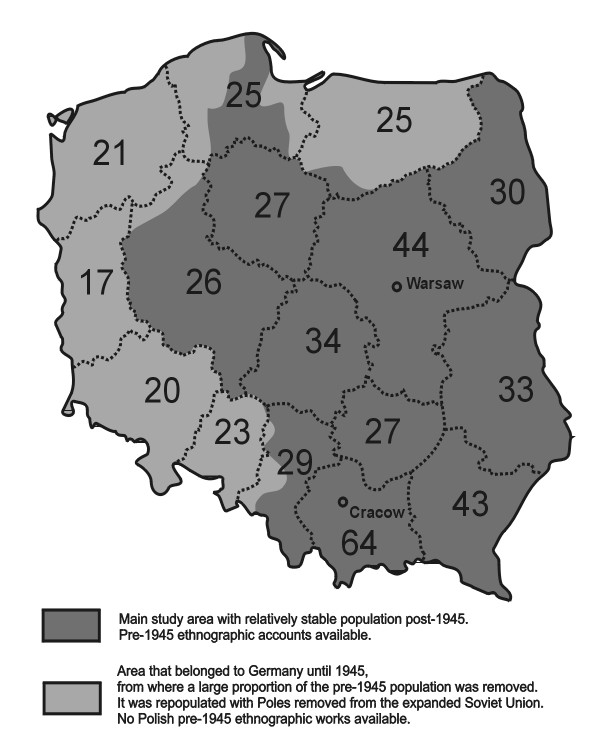
The number of wild plant folk species consumed in various regions of Poland.

Some records were not possible to identify on the species level, but only on the genus level. Thus a methodological problem arose, how to count numbers of species of edible plants, so that we, on the one hand, do not count the same species twice (e.g. as *Sonchus arvensis *and as *Sonchus *sp.), and on the other hand, do not underestimate the large diverisity of species contained in some folk species, e.g. *Rubus *or *Crataegus*, where several species are grouped under one folk name. Because of this issue we applied two measures of diversity. One was the number of folk species recorded (e.g. the two species of oak as one taxon, the many species of *Rubus *sect. *Rubus *as one taxon, but each *Vaccinium *species separately, as they have different folk names). This measure was applied to compare the numbers of taxa used in different regions (Fig. 2). On the other hand when we summarized the number of all species used in the whole country, we counted all the botanical species separately, e.g. *Quercus *as two species *Q. robur *and *Q. petraea*. In this calculation we also included the taxa identified to the genus level if no botanical species from this genus were recorded (e.g. *Galeopsis *sp.), and counted them as one species, but did not include records for *Sonchus *sp., nor *Malva *sp., as the use of some species of these two genera had already been identified. However we counted the record for *Ribes *sp. as a separate species, as it definitely concerned a different species than *Ribes nigrum*, recorded elsewhere.

Latin names of plants are listed according to Flora Europaea [71], and main synonyms are given including the name in the current checklist of Polish vascular plants of Poland [23]. Polish names of plants were also included, both official names from the checklist (**ON**) and local vernacular folk names (**LN**). Due to the great richness of variants of the local names, only the main ones or those contained in the cited works were used. Many of the local names are not unique to the given species and may refer to a few taxa, e.g. *oset*, for *Carduus *spp. and *Cirsium *spp. When official and local names were the same, we used the **ON/LN **symbol.

The list of wild plants used as food in Poland (see Appendix) is grouped into alphabetically sorted families. Each entry contains information in the following pattern:

***Latin name ***(syn. *Latin synonim*). **ON**: *official name*. **LN**: *local name*. **Part of the plant used 1**: means of consumption, approximate time when last used, region codes [references]; **Part of the plant used 2**: means of consumption, approximate time when last used, region codes [references]. **NOTE**. Taxonomic issues and other non-standard comments, e.g. more detailed description of preparation methods.

When literature referred to a folk species containing two or more species, the folk and latin name were written as follows: **folk species name 'FOLK SPECIES' (= *Latin names*)**.

In order to keep the list concise and to not inundate foreign readers with little known local geographic terms, the geographic location of use was given only on the regional level. The present administrative division of Poland into 16 regions called *województwo *was applied. The names of regions were coded as follows:

Ds – Dolnośląskie, Kp – Kujawsko-Pomorskie, Ls – Lubuskie, Lu – Lubelskie, Łd – Łódzkie, Mp – Małopolskie, Mz – Mazowieckie, Op – Opolskie, Pk – Podkarpackie, Pm – Pomorskie, Ps – Podlaskie, Sl – Śląskie, Sw – Świętokrzyskie, Wm – Warmińsko-Mazurskie, Wp – Wielkopolskie, Zp – Zachodniopomorskie (Fig. 1).

We did not include information on the collection time as it was rarely mentioned in the literature and it usually falls within two categories, i.e. green parts of plants in spring (March – June) and fruits in their ripening time (July–October).

When referring to the maps in the Ethnographic Atlas of Poland [32-34], we also quoted the map number after the colon, e.g. " [[33]:311]".

As some other authors [18], we use the classic term 'wild' in this review to refer to non-cultivated plants gathered in the field, including alien spontaneously occurring plants. In the case of species which are both cultivated and wild we have taken into account only records of the collection of non-cultivated individuals, e.g. in the case of *Malus*, *Pyrus*, *Rubus*, *Ribes *and *Armoracia rusticana*, which once cultivated, now occurs as an established ruderal weed and is rather collected from the wild than grown.

In this review we did not apply quantification of the cultural importance of a species based solely on the number of reports, applied in some similar works [18], as the number of literature sources was quite small and the amount of information they contained was very uneven. That is why we based our final rating of the intensity of use on the number of reports, and their geographical distribution as well as our weighing of the importance of particular papers. Here the data from the Ethnographic Atlas of Poland [31-35] were taken into account in the first place, as they came from a grid of 380 villages dispersed evenly throughout the country. As 'commonly gathered species' we treated the species whose collection was documented from the 1960s or later, in at least half of the sixteen regions of Poland.

## Results

The use of 112 species of vascular plants as food, seasoning or beverage has been recorded in the Polish countryside since the 18^th ^century. They belong to 81 genera from 39 plant families. The list includes 20 species of trees, 23 species of shrubs (including 6 species of dwarf shrubs), 49 species of perennials, 3 species of biennials and 16 species of annuals. The largest number of species (nearly half) belongs to the category of green of vegetables – 53 species. The ripe fruits (both fleshy and dry) and seeds of 43 species have been consumed as well as the underground parts of 10 species. Various parts of 16 species have been used as seasoning, and 13 species have been used as ingredients of bread.

There is large geographical variation in the number of species used (Fig. 2). The largest number of folk species – 64, was recorded in the *Małopolskie *region, the hilliest area of Poland. This is much more than in the next region, *Mazowieckie*, with 44 species and *Podkarpackie*, with 43 species. The lowest numbers of folk species – 17, 20 and 21, were recorded in the three westernmost regions, where most of the pre-World War II population was moved to Germany and most of the present inhabitants are Poles moved from the eastern outskirts of pre-war Poland annexed by Soviet Union. Generally speaking, the south-eastern half of Poland has much stronger traditions of using wild plants as food than the north-western half.

It must be stressed that the analyzed literature documents an absolutely dramatic decrease in the use of wild plants as food. Only the use of 16 species as green vegetables (a lot of them solely as children's snacks) was recorded around the 1960s or later, which constitutes only 30% of all the recorded green vegetables. Out of them it is only *Rumex acetosa *that has remained a part of the everyday cuisine. In the 19^th ^century most of the recorded green vegetables were already treated as famine food or the food of the poor.

. Less of the traditional heritage has been lost in the case of fruits and seasoning. Thirty species of fruits (70%) are or were recently (1960s or later) consumed, including all the fleshy-fruited species, except *Empetrum nigrum *and *Maianthemum bifolium*, whereas the forgotten species are usually the ones with dry fruits (e.g. *Bromus secalinus *and *Glyceria fluitans*). Most of the traditional condiments (11 species, 69%) are also still used or remembered from recent past.

The list of wild edible plants which are still commonly gathered includes only two green vegetables (*Rumex acetosa *leaves for soups and *Oxalis acetosella *as children's snack), 15 folk species of fruits and seeds (*Crataegus *spp., *Corylus avellana*, *Fagus sylvatica*, *Fragaria vesca*, *Malus domestica*, *Prunus spinosa*, *Pyrus *spp., *Rosa canina*, *Rubus idaeus*, *Rubus *sect. *Rubus*, *Sambucus nigra*, *Vaccinium myrtillus*, *V. oxycoccos*, *V. uliginosum*, *V. vitis-idaea*) and four taxa used for seasoning or as preservatives (*Armoracia rusticana *root and leaves, *Carum carvi *seeds, *Juniperus communis *pseudo-fruits and *Quercus *spp. leaves). The use of other species is either forgotten or very rare.

### Green vegetables

Green vegetables, which include plants whose green parts such as leaves, stalks or unripe fruits are eaten raw or after special preparation (cooking, frying, etc.), excluding plants used in small quantities only as seasoning, constitute the largest use category, as the use of 53 species (49% of all used species) was recorded. The most represented families are Asteraceae (9 species), Brassicaceae (5 species), Polygonaceae, Pinaceae (both 4 species) and Chenopodiaceae (3 species). Most of the recorded green vegetables are plants, which were eaten in times of scarcity, usually as mixed potherb, often with an admixture of potatoes, *kasza *(cracked buckwheat or cracked cereals), butter, milk or cream. The traditional name for such potherb was *jarmuż *[60,62] or, in the Carpathians, where it was particularly popular, *warmuz *[1,45,50]. The use of all the Asteraceae disappeared at the beginning of the 20^th ^century, with the exception of *Taraxacum*, occasionally used later. It should be noted that the use of Asteraceae was restricted mainly to the Carpathians, especially to their western part (Mp, Sl). The only widely used green vegetables, which are still commonly used in nutrition are *Rumex acetosa *(leaves used to make soup), and to a lesser extent *Pinus sylvestris *(shoots for making syrup). Other presently used green vegetables are mainly children's snacks consumed raw. *Chenopodium album*, *Urtica dioica *and *U. urens *used to be other important potherb plants, however their use has gradually disappeared, so that already around World War II they were seen mainly as famine plants and their use was restricted to poor or elderly people, or individuals who particularly liked them.

Lacto-fermenting wild vegetables, once very widespread among Slavs [1,26,27] have completely disappeared from the countryside. The last lacto-fermented wild vegetable in Poland was hogweed *Heracleum sphondylium*. The use of fermented leaves and stalks of *H. sphondylium *was first mentioned by Marcin z Urzędowa in 1595 [72]: "Whoever eats hogweed, moistens his living. (...) When they make it sour in the Polish way, it is good to drink in fevers, thirst, as it alleviates thirst and cholera and it induces greed for food with its spice. (...) Garnished with egg and butter, it is good to eat on the days when they do not eat meat soup, as it works in the same way." The use of this plant in Poland and Lithuania was also mentioned by Gerarde (as *Spondylium*) in 1597 [73]. Another early account comes from Syrennius [74]: "Hogweed is familiar to everyone in our country, in Ruthenia, Lithuania and Żmudź. (...) It is useful as medicine and for food is very tasty. Both roots and leaves.

However the root is more useful as medicine and leaves as food. (...) Leaves are commonly gathered in May. (...) Soup made with it, as it is made in our country, Lithuania and Ruthenia, is tasty and graceful. Either cooked on its own or with chicken or other ingredients such as eggs, cream, millet." Hogweed was the main lacto-fermented soup of Slavs, the young leaves and stalks were covered with warm water and left for a few days to become sour [1].

According to a 17th century menu hogweed soup was served every Wednesday during the period of Lent for the professors of Jagiellonian University in Cracow and they also ate it as the main soup at Easter [75]. In the 18^th ^century it was already a rare food for poorer people, being replaced by beetroot soup, which took the name *barszcz *earlier attributed to hogweed [26], as Ładowski [76] wrote that "the vulgar people use hogweed to make a soup called *Barszcz*". Jundziłł [77] gave a description of its use in Lithuania, which was probably the same as the use in Poland: "they collect young leaves, ferment them in the same fashion as other vegetables and they are frequently eaten by village people. Or, dried in the shade like celery, they are kept for further use." The sudden decline of its use in the 18th century is documented by the fact that hogweed soup is not mentioned by Kluk [25]. According to Rostafiński hogweed soup stopped being made in Poland in the 18th or 19th century and the last record of its use in adjacent Lithuania comes from 1845 [26]. However Moszyński witnessed it still being made in Russia in the 20^th ^century [27]. Surprising new data on the use of hogweed soup in Poland were found during the research for this review. According to Professor Adam Zając from the Institute of Botany, Jagiellonian University in Cracow (personal communication) this dish was used by his grandmother, Anna Tomiak (born 1880) up to the 1940s or 1950s, in the village of Straconka (now a part of the city of Bielsko-Biała), in the Beskid Mały Mountains (Sl). She placed stalks and leaves in a jar, covered them with water and left them for a few days, then she cooked soup with them. Another record of the traditonal use of *H. sphondylium *in the last century comes from the village of Łapsze Niże (Spisz area, Mp), where it was used until the 1920s, mixed with other plants as potherb or made into "sour soup" [49].

### Fruits and seeds

Out of 43 species whose use was recorded in the category of fruits and seeds (excluding species used only as seasoning), nearly half, i.e. 18 species belonged to the Rosaceae family. Other important families are Ericaceae with 5 species, as well as Poaceae with 4 species and Fagaceae with 3 species.

The fruits most commonly collected from the wild include *Vaccinium myrtillus*, *V. vitis-idaea*, *V. oxycoccos*, *Rubus idaeus*, *Rubus *spp. from section *Rubus *(particularly *R. hirtus*, *R. nessensis *and *R. plicatus*), *Sambucus nigra *and *Fragaria vesca*, and to a much lesser extent *Prunus spinosa*, *Sorbus aucuparia*, *Crataegus *spp., *Vaccinium uliginosum *and *Corylus avellana*.

In the past most fruits were eaten fresh or dried, whereas grass seeds (*Glyceria *spp. and *Bromus secalinus*) were used to make gruel or bread. In the second half of the 20^th ^preserving soft fruits in the form of jams, wines and pasteurized compotes became popular. However within the last few years it has been in decline due to the society's growing affluence.

### Underground parts

The use of underground parts of plants (roots, rhizomes, bulbs) was recorded only for 10 species. *Elymus repens *rhizomes were particularly widely used. They were dried, ground and used to make soup, gruel or bread. Less common, mainly in the northern part of Poland, was the digging out of *Pastinaca sativa *roots. Use of sweet rhizomes of *Polypodium vulgare *survived until the 20^th ^century only as a children's and shepherd's snack.

### Seasoning and preservatives

Out of the 16 species used as seasoning or preservatives nearly half, i.e. 6 species (37%), belong to the Lamiaceae. Although the Lamiaceae are the most represented family, none of the species from this family are commonly used nowadays. The only wild plants used presently on a larger scale as seasoning are *Carum carvi *(seeds), *Armoracia rusticana *(grated roots, leaves used as a fragrant base for baking bread), *Acorus calamus *(leaves used as a fragrant base for baking bread) and *Quercus robur *and *Q. petraea *leaves (added to pickled cucumbers and sometimes used as a base for baking bread).

Placing a large leaf under baking bread is a very widespread phenomenon. Such activity both prevents bread from sticking and gives it a unique flavour often recalled by people interviewed by ethnographers. This practice is in decline along with the disappearing tradition of making bread in every house, but as it was still alive in the 1960s, it has probably partially survived up to the present. The use of particular species shows strong geographical patterns. The use of cabbage leaves is widespread throughout Poland, whereas the use of wild plants leaves is more local, with *Armoracia rusticana *mainly in central and NE Poland, *Acorus calamus *in NE Poland, and *Acer platanoides*, *A. pseudoplatanus *and *Quercus robur *used in just a few villages.

Poles generally use few wild plants as seasoning except for making bread, in which case *Carum carvi *seed have been universally used, and for making sauerkraut *kiszona kapusta *and lacto-fermented cucumbers *ogórki kiszone *(*ogórki kwaszone*). *Carum carvi *seeds and fruits of the feral forms of *Malus domestica *have been traditionally added to sauerkraut, whereas cucumbers are fermented with garlic, *Anethum graveolens *umbels (cultivated), *Armoracia rusticana *root (dug out from the wild) and obligatorily at least one leaf of the following species: *Quercus robur *or *Q. petraea *(wild), *Cerasus vulgaris *(cultivated) and *Ribes nigrum *(cultivated) [30,65].

### Beverages

The main kind of herbal drink, and the only one drunk, up to the 20^th ^century, on a nearly every day basis, during the cold season, in the Polish countryside was the *Tilia *flower infusion. Another common drink, a coffee substitute among peasants, was an infusion of roasted acorns.

In spring tree sap was drunk, mainly fresh, only extremely rarely concentrated or fermented. This mainly concerned the sap from *Betula pendula *and *B. pubescens*, and to a lesser extent from *Acer pseudoplatanus *and *A. platanoides*. Drinking tree sap was gradually disappearing from the Polish countryside in the 19^th ^and the beginning of the 20^th ^century, becoming nearly obsolete, however is now reviving as a part of health food fashion.

Using juniper "berries" as the main ingredient of beer was very widespread in northern, central and north-eastern Poland, but nowadays survived only in the Kurpie area (central-NE Poland, Mz), where it is called *psiwo jałowcowe *or *psiwo kozicowe*.

Making juices, wine and, to a much lesser extent, liqueurs out of wild fruits seems to be mainly a 20^th ^century fashion, rarer in earlier times, but an extremely widespread activity in the countryside in the second half of the 20^th ^century in the Communist period (1945–1989), now diminished by the increasing affluence of society. The main kinds of fruits used for this purpose are *Rubus idaeus *(also added to black tea), *Rubus *sect. *Rubus*, *Sambucus nigra*, *Prunus spinosa*, *Rosa canina*, *Crataegus *spp. and *Sorbus aucuparia*.

### Bread ingredients

The seeds *of Glyceria fluitans *were used to make bread which was highly praised in the past [65], but the use of this plant died out completely at the beginning of the 20^th ^century. Other plants were used as famine additions admixed to ordinary leven bread or simple flatbread, or, only in extreme situations, used to make flatbread composed solely of wild plants. The use of the following plants was recorded: *Elymus repens *rhizomes, *Betula *and *Tilia *cambium, *Corylus avellana *inflorescences and *Pinus sylvestris *needles as well as ground fruits of *Quercus robur, Q. petraea, Fagus sylvatica, Bromus secalinus *and *Malva neglecta*, and ground seeds of *Vicia *spp. and *Calluna vulgaris*.

### Children's snacks

Some wild plants, such as *Capsella bursa-pastoris *and *Malva sylvestris *unripe seeds, *Scirpus sylvaticus *and *Dactylis glomerata *stem bases, *Padus avium*, *Cerasus avium and Maianthemum bifolium *fruits, *Campanula persicifolia *flowers, *Polypodium vulgare *rhizomes and *Phyteuma spicatum *roots have been used almost exclusively by children, but this may be a relic of a more widespread use.

## Discussion

The presented list of species is not very long. The use of 112 species was recorded (3.7% of the flora), most of them as obsolete famine foods and children's snacks. Out of these only 51 species have been used since the 1960s. Of them the list of still commonly collected wild edible plants includes only two green vegetables, 15 folk species of fruits and three taxa used for seasoning or as preservatives. The use of other species is either forgotten or very rare. So within the last 100 years a marked shift has occurred, from collecting a variety of plant parts, including leaves of common ruderal and grassland plants cooked for potherb (especially *Urtica *spp., *Chenopodium album*, *Atriplex *spp.), and starch-rich famine plants (*Elymus repens*, *Quercus *spp.), to collecting mainly forest and forest edge fruits, and a few species of seasoning. There are many species whose use was reported from the Ukraine, Belarus or Russia [1,4,27], which have not been recorded as food plants within the present territory of Poland within the last few hundred years (e.g. *Arum *spp., *Orchis *spp., *Calla palustris*, *Bunias orientalis*, *Nymphaea alba*, *Chaerophyllum bulbosum*, *Polygonum bistorta*, *Tragopogon pratensis*, *Angelica sylvestris*). Many species consumed during famine or food scarcity in the 19th century and during World War I in Germany and Austria [1], and common in Poland, e.g. *Aegopodium podagraria *and *Alliaria petiolata*, also do not appear in Polish culinary ethnographic literature either. The use of some of these species might have become obsolete before ethnographic studies began, e.g. *Aegopodium podagraria *leaves used for potherb in medieval times [66]. Looking at the use of potherb plants, four categories of plants can be distinguished. Firstly, plants which were probably used as potherb only before written records (e.g. *Aegopodium podagraria*, *Angelica sylvestris*, *Alliaria petiolata*). Secondly, plants whose common use stopped between the 18^th ^century and the beginning of the 20^th ^century (e.g. *Alchemilla *spp., *Cirsium rivulare*, *C. oleraceum*, *Glechoma hederacea*, *Malva *spp., *Heracleum sphondylium*, *Ranunculus ficaria*, *Sonchus *spp., *Symphytum officinale*, *Taraxacum *spp., *Tragopogon *spp., *Tussilago farfara*, *Polygonum *spp., *Pulmonaria obscura*) with only small traces of their use recorded. Thirdly, plants whose use has practically died out, but is vividly remembered by a large proportion of the population (*Urtica *spp., *Chenopodium *spp., *Atriplex *spp. and *Oxalis acetosella*). Fourthly, plants which are still in use today as potherbs, i.e. *Rumex acetosa *and *R. acetosella*. Strangely, there seems to be no clear explanation why the species disappeared from cuisine in such an order.

It must be noted that the number of wild vascular plant food species in Poland has been, at least within the last 200 years, extremely low, compared to some regions of Southern and Eastern Europe. This issue has already been raised by Moszyński [27] and confirmed by the Polish Ethnographic Atlas [31]. For comparison, in Spain over four times more wild culinary plants were recorded (419 species compared to 112 in Poland), which constitutes 6% of Spain's flora (compared to 3.7% of Polish flora). In Bosnia and Herzegovina, a country six times as small, but with a number of plant species similar to Poland, the use of three times as many (308) plant species was recorded [19]. In Sicily, an island fourteen times as small, whose population is eight times as small and which has the same number of plant species as Poland, 188 species wild edible plants were found, which is 6.2% of the flora, compared to 3.7% for Poland [78]. One small part of Catalonia in Spain has a list of edible plants containing 75 species, nearly as long as the list for the whole of Poland, including species whose use has been long obsolete [8]. Other Mediterranean regions have been also repeatedly reported to have high number of edible plants used, e.g. the region of Madrid – 123 species [9], Campoo (Spain) – 60 species [10] and one area in Italy with over seven thousand inhabitants – 44 species [15]. If only the species still used in the 1960s or later are taken into account (around 50), the whole of Poland has less species than one small Mediterranean region! Another interesting comparison which can be drawn comes from Italy. Picchi & Pieroni [79] listed over 150 species of herbs used in traditional Italian cooking. Again, more species than the presented list for Poland, although their book does not include staple plants, fruit trees, roots etc. In one village of southern Italy, Castelmezzano, with less than a thousand inhabitants, the use of 60 species of edible plants was recorded [14], which is nearly the maximum number of edible plants recorded in a Polish region (Fig. 2) and double the maximum number of edible species recorded in a local ethnobotanical study in Poland [53].

Two factors may be responsible for this contrast between the rich heritage of using wild edible in southern Europe and a relative lack of it in the north. One reason is the gradual impoverishment of floras towards the north. In northern countries like Poland the flora is poorer, hence the choice of species is poorer as well. Polish flora has 3000 species compared to around 6700 species in Italy [80] or 7000 in Spain [18]. On the other hand in just two small regions in Cyprus, an island with a flora of around 2000 species (less than the Polish flora), the use of as many as 78 species of wild edible plants was recorded [16]. Thus the other factor, the culinary habits, must be more important. Most edible plant species used in the Mediterranean are appetizers or spices in soups, or ingredients of omelettes, salads and beverages, not staple foods [e.g. [15,17,18,79]]. Many of them can be found as common plants in Poland. The Polish countryside has very bland food and does not have a strong tradition of adding locally collected spices. Even *Thymus *spp. and *Origanum vulgare*, extremely common in some areas of Poland and used as culinary herbs in other countries of Europe, have hardly ever been used in Polish cooking as spices, although they are often listed by ethnographic sources as medicinal plants used as infusions throughout Poland [55,56,66]. The primary reason for difference in attitudes towards herbs between Poland and the Mediterranean is climate. In warmer climates the addition of herbs to meats, dairy and sauces kept them from going off, whereas in the Polish temperate climate there is less need for this. Hence "pure", refined foods like white sugar, white bread and pure good quality meat were most highly prized, and wild plants, apart from fruits and mushrooms, were associated with times of famine and seasonal spring food shortages.

**Figure 3:**
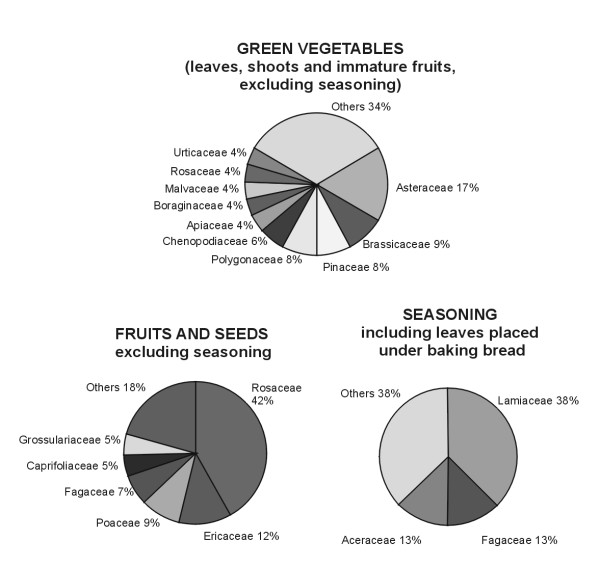
Botanical families cited for the major food categories. All families represented by ate least two species per category were included.

The proportion of families in the wild plants consumed in Poland (Fig. 3) is similar to this of Mediterranean countries. Similarly to Spain [18] the most important families of edible fruits are Rosaceae, Ericaceae and Fagaceae, the majority of species used for seasoning comes from Lamiaceae, and the best represented family in the category of green vegetables are Asteraceae (however the use of most Asteraceae in Poland is obsolete). On the other hand the main difference is the nearly complete absence of the use of Liliaceae species in Poland, whereas among Mediterreanean edible plants they constitute one of the most important groups of plants [17,18].

Probably due to the extremely low endemism levels of the Polish flora, most of the plants recorded as edible in Poland are known to be used in a similar way in other countries [1-7]. The exceptions are three species used for famine potherb: *Cirsium rivulare*, *Euphorbia peplus *and *Lemna minor *and one species, *Scirpus sylvaticus*, used as a children's snack. Three of these species have never been listed as edible in any ethnobotanical papers concerning wild food, and *L. minor *was mentioned only by one author [81].

In this review, with a few exceptions such as master's theses, we did not include unpublished material on gathering wild plants, which is stored in some ethnographic institutions in Poland (universities, Polskie Towarzystwo Ludoznawcze in Wrocław and the office of the Ethnographic Atlas of Poland in Cieszyn), as this requires further, extensive study. The archives contain mainly answers to questionnaires used in the research for the Ethnographic Atlas of Poland and notes from field interviews. The maps published for the Ethnographic Atlas of Poland summarized the use of the most important and widely used species [32,33] and the use of some species, using the data from the archives, was discussed by Paluch [65], Bohdanowicz [31] and Jędrusik [35], but much data is still waiting to be summarized and published, including the manuscript of the seventh volume of the Atlas [34].

Further special ethnobotanical research is needed both in ethnographic archives and in the field to record the lesser known species of edible plants, often neglected by ethnographers. A feature which is very characteristic of Polish science is the lack of the recognition of ethnobotany as a separate subject. Most ethnobotanical studies were carried out by ethnographers with somewhat limited botanical knowledge, usually restricted to the level of botanical genera and folk species. On the other hand most botanists are not aware of the methods applied by ethnobotany and sometimes are not aware that valuable ethnobotanical studies are carried out in their university, only outside the botany department. A good example here is the case of *Mentha*. Most ethnographic papers do not distinguish between various mint species, labelling them *Mentha *sp. [56] or *Mentha piperita *[35,55]. Only two more botanically aware authors noticed that what is collected from the wild can be two native species – *Mentha arvensis *[50] and *M. longifolia *[36], which have been until recently completely neglected in ethnobotanical research.

Culinary habits are never static. Within the last few years a strong revival in the use of wild plants can be observed. Local food producers are trying to popularise, rediscover or even invent "local products" which can be sold to tourists. This process had already started in the 1980s when villagers in Łapsze Niżne, in the Carpathians, sold *Abies alba *shoots syrup to tourists as a 'local speciality' [49]. Juniper beer has recently been rediscovered for commercial purposes in the Kurpie area (Tomasz Madej, spoken communication). Also, the media popularize the use of wild plants in cooking. A good example of their influence is a surge in interest in the culinary use of *Allium ursinum*. The famous culinary TV presenter, Robert Makłowicz, showed it sold in a vegetable market in the Ukraine in the TV programme titled *Podróże kulinarne Roberta Makłowicza *and a few popular publications on this species were published by the first author (Ł.Ł.) e.g. in the monthly magazines *Wróżka *and *Ogrody*. Within the last few years pasteurized birch sap, dried *Allium ursinum *and *Urtica dioica *leaves and oak coffee have appeared in health food shops heralding a period of increased interest in wild foods. The strong influence of the media on the collection of wild products was already observed by in the 1970s and 1980s [47,49]. In that period publications in the women's magazine *Przyjaciółka *and popular culinary guides by Irena Gumowska were particularly influential [e.g. [82,83]]. The presented list of plants collected in Poland will allow the drawing of a clear boundary between what is a traditionally collected plant and what is a borrowing from another nation's culinary habits or a rediscovery of plants used in prehistory or in early Polish history. It will also help future researchers to focus on the more overlooked or confused taxa.

## Conclusion

1. At least 112 species of plants, belonging to 81 genera and 39 families have been used to make food and drink in the Polish countryside.

2. Only half of these species have been used to some extent since the 1960s, the usage of the other half stopped between the 18^th ^century and the 1960s. The utilization of wild fruits is still continued, whereas it is the category of wild green vegetables, which has been almost completely forgotten.

3. The proportion of flora utilized as edible plants is much lower compared to the countries of southern Europe, where relevant ethnobotanical research was carried.

4. The proportion of families used is similar to that of the Mediterranean countries, with the exception of Liliaceae, which are little used in Poland.

5. Further special ethnobotanical research is needed both in ethnographic archives and in the field to find the lesser known species of edible plants, often neglected by ethnographers.

## Competing interests

The author(s) declare that they have no competing interests.

## Authors' contributions

The article was initiated by both authors, who started searching the literature together and made the preliminary list of plants. The whole search of unpublished archival materials, the final preparation of the manuscript, and the literature search in the second stage were done by Ł. Łuczaj. Both authors read and approved the final version of the manuscript.

## Appendix. List of species

### Aceraceae

***Acer platanoides *L. ON**: *klon zwyczajny, klon pospolity*. **LN**: *klon*. **Sap**: fresh, rarely used, at least until the 1960s, Pk, Ps, Wm, Wp, Zp [[31,33]:311], until the early 20^th ^century, Mp [[33]:311]; fermented to make beer by settlers from the present area of Lithuania and Belarus, in the mid-20^th ^century, probably no longer used, in Wrzosy, Wm, reports about drinking fermented sap, still in the 1960s, came also from Zp, Ls, Ds [[31,33]:311]. **Cambium**: eaten raw as a children's snack, until the early 20^th ^century, Mz [52].**Fruits (mature and immature)**: eaten raw as a children's snack, until the early 20^th ^century, Mz [52]. **Opening leaf buds**: fermented in a wooden container to make soup, until the early 20^th ^century, Mz [52]. **Leaves**: put in the oven under baking bread, partly to prevent the bread from sticking and partly to flavour the bread, at least until the mid-20^th ^century, probably still used, Mp, Lu, Zp [[32]:265].

***Acer pseudoplatanus *L. ON**: *klon jawor*. **LN**: *jawor*. **Sap**: fresh; at least until the 1960s, Mp, Pk [[31,33]:311]. **Leaf buds**: raw, eaten by shepherds, until the late 19^th ^century, Mp [27]. **Leaves**: put in the oven under baking bread, partly to prevent the bread from sticking and partly to flavour the bread, at least until the mid-20^th ^century, probably still used, Pk [[32]:265].

### Apiaceae

***Carum carvi *L. ON**: *kminek zwyczajny*. **LN**: *kminek*, *kmin*, *warmuz*. **Seeds**: as a spice, especially for bread, sauerkraut or soft cheese, widely collected from the wild until the 20^th ^century, now the job is done mainly by herbalist companies, very rarely by private individuals, commonly used in modern cooking, available in shops, Mp [35,49], Pk [65], Łd [54], Mz [35,53], Pm [35,62], Sl, Lu, Ps, Kp [35]. **Young plants**: boiled, in a famine potherb called *warmus *or *warmuz*, a mixture of wild plant leaves, served with potatoes and butter, used until the late 19^th ^century, Mp [42,45,65].

***Heracleum sphondylium *L. s.l. ON**: *barszcz zwyczajny*. **LN**: *barszcz*. **Leaves and flowering stalks**: collected in spring to be cooked to make soup, often lacto-fermented before cooking, used commonly until the 17–18^th ^century throughout the country [1,26,27], no longer used, used extremely rarely until the 1920s, Mp [49] and the 1950s, Sl (Adam Zając, personal communication). **NOTE**. For more details see the Results section.

***Pastinaca sativa *L. ON**: *pasternak zwyczajny*. **LN**: *pasternak*. **Roots**: used in various cooked or fried foods, dug out in late autumn, until the early 19^th ^century commonly grown as a vegetable, later the roots sometimes collected from the wild or semi-wild state and used as a vegetable [[1,31,34]:360].

### Araceae

***Acorus calamus *L. ON**: *tatarak zwyczajny*. **LN**: *tatarak*. **Stems**: inner parts of young shoots eaten raw, until the early 20^th ^century, Mz [52], until now sometimes used as children's snack throughout the country, e.g. Pm [62]. **Leaves**: put in the oven under baking bread, partly to prevent the bread from sticking and partly to flavour the bread, still used, Mz, Ps, Wm, Zp [[32]:265, [36]].

### Asteraceae

***Carlina acaulis *L. ON**: *dziewięćsił bezłodygowy*. **LN**: *dziewięćsił*, *dziewięcioł*, *rzepik*. **Roots**: means of preparation not specified, until the mid-19^th ^century, Mp [41]. **Receptacles**: means of preparation not specified, until the turn of the 19^th ^and 20^th ^century, eaten by child shepherds, Mp [50], Sl [64]. **NOTE**. Although the former publication [50] refers to *C. vulgaris*, the identification was verified to *C. acaulis *in the telephone conversation with the author of the report (Urszula Janicka-Krzywda).

***Carlina vulgaris *L. ON**: *dziewięćsił pospolity*. **Unspecified parts**: as famine food, until the early 20^th ^century, Mp [48]. **NOTE**. This reference to *C. vulgaris *might be a mistake and in fact describe the use of *C. acaulis *(equally common in the Carpathians and larger) or at least both of the species indiscriminately.

***Centaurea cyanus *L. ON/LN**: *chaber bławatek*. **LN**: *chaber*, *bławat*, *bławatek*. **Flowers**: combined with sugar to make wine, commonly used until the mid-20^th ^century, but now nearly forgotten, Pk [55,65], Ps [65]; used to dye vinegar, until the 18th century [25].

***Cichorium intybus *L. ON/LN**: *cykoria podróżnik*. **Leaves**: preparation methods not specified, used in the 18th century [25], eaten in the late 19^th ^century during famine, Mp [1]. **Roots**: roasted, as a coffee substitute, also used as a vegetable (without the bitter inner part), in the 18^th ^century [25].

**folk species ' *OSET' *(= *Cirsium *sp. pl. &*Carduus *sp. pl.). Young shoots**: boiled in soup, mainly Mp, also Łd [[31,33]:357]; boiled as potherb, mainly Mp, also Lu [[31,34]:357], chopped and eaten raw, Pk, Łd [[31,34]:357]; used until the turn of the 19^th ^and 20^th ^century. **NOTE**. In folk taxonomy there is no distinction between *Cirsium *and *Carduus*. Probably several local species of these genera were used for famine potherb, maybe including the common arable weed ***Cirsium arvense *(L.) Scop**.), however no voucher specimens are available, so the taxon *oset *was not included in calculations, due to a possible overlap with *Cirsium rivulare *and *C. olearceum*.

***Cirsium oleraceum *Scop. ON**: *ostrożeń warzywny*. **LN**: *oset*, *czarcie żebro*. **Leaves**: scalded and fried with lard, butter, cream, flour or eggs, Łd [54]; the leaves and roots were boiled with milk as soup, Łd [54]; boiled in a famine potherb called *warmuz*, a mixture of wild plant leaves served with potatoes and butter, Mp [45]; used until the turn of the 19^th ^and 20^th ^century, only as famine food, Mp [45], Łd [54]. **Roots**: boiled together with the leaves and milk as soup, used until the turn of the 19^th ^and 20^th ^century, only as famine food, Łd [54].

***Cirsium rivulare *All. ON**: *ostrożeń łąkowy*. **LN**: *scérbok*, *oset*. **Leaves**: young leaves, before flowering, boiled, used for *warmuz *potherb, only as famine food, served with boiled potatoes, oats flour, butter or milk, used until the late 19^th ^century, Mp [45]. **NOTE**. A reference to scalded *szczerboc *leaves, eaten as famine food together with scalded *macierzanka *leaves (probably *Thymus pulegioides*) in the Gorlice area probably pertains to *C. rivulare *as well, Mp [46].

***Sonchus arvensis *L. ON**: *mlecz polny*. **LN**: *mlecz*. **Green parts**: eaten "as lettuce", until the beginning of the 20^th ^century, Mp [49]. **NOTE**. Due to the fact that both *Sonchus *and *Taraxacum *are called *mlecz*, this reference must be taken with caution.

***Sonchus *sp. ON/LN**: *mlecz*. **Green parts**: in the past boiled in mixed potherb (*jarmuż*), Kp [61]. **NOTE**. Due to the fact that both *Sonchus *and *Taraxacum *are commonly called *mlecz*, this reference may refer to *Taraxacum *as well.

***Taraxacum *sp. pl. ON/LN**: *mniszek*. **LN**: *mlecz, dmuchawiec, pępawa*. **Inflorescences**: commonly used to make syrup or wine e.g. [56], probably in all regions, but this may be a 20^th ^century fashion popularized by the media. **Leaves**: boiled and drained leaves were mixed with milk, whey or boiled potatoes, until the turn of 19^th ^and 20^th ^century, as famine food, Mp [45], Mz [65], nowadays sometimes used for salads as a part of a health food fashion (personal observation). **Stalks**: known as *pępawa*, roasted on hot stones by shepherds, until the early 20^th ^century, Mp [50]. **NOTE**. *Taraxacum *and *Sonchus *may be confused due to the same folk name.

***Tussilago farfara *L. ON**: *podbiał pospolity*. **LN**: *podbiał*, *podbielina*. **Leaves**: boiled, used as famine potherb, until the beginning of the 20^th ^century, Mp [45].

### Berberidaceae

***Berberis vulgaris *L. ON**: *berberys zwyczajny*, *berberys pospolity*. **LN**: *berberys*. **Fruits**: raw and preserves (jam, juice or wine), commonly collected in some areas in the 1960s, probably still used, mainly Lu, Sw [[31,34]:367] and Mz [[53,65,34]:367], sporadically Mp [49], Pk, Wp [[34]:367]; condiment, with cabbage dishes, instead of vinegar, until the mid-20^th ^century, Mz [53], Łd [65]; liqueur, in the 1960s, Mz [[34]:367].

### Betulaceae

**folk species 'BRZOZA' (= *Betula *sp. pl.) ON/LN**: *brzoza*. In folk taxonomy the two native tree species of birch, ***Betula pendula *Roth **(syn. *B. verrucosa *Ehrh.) and ***Betula pubescens *Ehrh**. are not distinguished and used indiscriminately, the former more often. **Sap**: fresh, used in all regions until the mid-20th century, most commonly in Pk, Lu, Mz, Ps, use strongly decreased throughout the 20^th ^century [[25,31,33]:311, [37,40,52,57,60,61,31]], but is now reviving as a curiosity or health food; boiled sap thickened with rye flour and milk, until the mid-20^th ^century, Mz [53]; concentrated into syrup used to sweeten drinks, until the early 20^th ^century, Mz [52]. **Cambium**: dried and ground, sporadically used as an ingredient of famine bread, until the early 20^th ^century, Mp [46], Mz [[33]:322, [52]], Pk [[33]:322]; scraped soft mucous parts, in early spring, fragmented, used "in a similar fashion to butter", until the early 20^th ^century, Mz [52]. **Leaf buds**: opening buds in spring, fermented in wooden containers, used to make soup, until the early 20^th ^century, Mz [52].

### Boraginaceae

***Pulmonaria obscura *L**. (syn. *P. officinalis *L. subsp. *obscura *(Dumort.) Murb.) **ON**: *miodunka ćma*. **LN**: *miodunka*, *swykołka*. **Leaves**: boiled, in potherb with other species, as famine food in spring, until the late 19^th ^century [1], e.g. Lu [40]. **NOTE**. Originally recorded as *P. officinalis*, however *P. officinalis *L. *sensu stricto *is not recorded in the area, being restricted to the western outskirts of Poland [67], so the above mentioned references probably refer to the closely related *Pulmonaria obscura *Dum.

***Symphytum officinale *L. ON**: *żywokost lekarski*. **LN**: *żywokost*. **Leaves**: as famine food, preparation method not specified, until the early 20^th ^century, Mp [48]; one informant reported using chopped leaves in traditional fritters in the Ojców area, Mp [36].

### Brassicaceae

***Armoracia rusticana *P. Gaertn., B. Mey. & Scherb**.(syn. *Armoracia lapathifolia *Gilib.) **ON**: *chrzan pospolity ***LN**: *chrzan*, *krzan*. **Roots**: raw, whole as a condiment, with pickled cucumbers, grated with chopped boiled eggs, soups or meat dishes, often used at Easter, still widely used, usually collected from the wild and not cultivated, as it now occurs as a difficult to eradicate weed, all regions [35,36,57,65]. **Leaves**: raw, put in the oven under baking bread, partly to prevent the bread from sticking and partly to flavour the bread, still widely used, commonly in Ps, Lu, Mz, Wm, rarely in Pk, Wp, Ds, Zp [[32]:265, [65]]; lacto-fermented with rye, then cooked with peas; Mz [53], until the mid-20^th ^century.

***Capsella bursa-pastoris *(L.)Medik. ON**: *tasznik pospolity*. **LN**: *tasznik*. **Unspecified parts**: mentioned by Moszyński [27] as one of the typical Slavic wild foods. **Fruits**: still widely known as a common children's snack, throughout the country, e.g. Pk (personal observation).

***Raphanus raphanistrum *L. ON/LN**: *rzodkiew świrzepa*. **LN**: *hodryk *(collectively with *Sinapis arvensis*). **Leaves**: boiled in a famine potherb called *warmuz*, until the late 19^th ^century, Mp, [45].

***Sinapis alba *L. ON/LN**: *gorczyca jasna*, *gorczyca biała*. **Leaves**: famine food until the late 19^th ^century, in an unspecified area [1].

***Sinapis arvensis *L. ON/LN**: *gorczyca polna*. **LN**: *ognicha*, *gorczyca*, *pszonak*, *hodryk*. **Leaves**: boiled or fried, as famine food, in mixed potherb, used until the early 20^th ^century, Mp [42], Mz [52,53], Łd [54]. **NOTE**. In Central Poland (Mz, Łd) young leaves were first scalded, squeezed out to get rid of bitterness and then fried with milk, cream or, in better times, with flour or eggs [53,54].

### Campanulaceae

***Campanula persicifolia *L. ON**: *dzwonek brzoskwiniolistny*. **LN**: *dzwonek*. **Flowers**: raw, eaten by children while picking *Vaccinium myrtillus *and *Fragaria vesca *fruits, at least until the mid-20^th ^century, Mz [53].

***Phyteuma spicatum *L. ON**: *zerwa kłosowa*. **LN**: *zajęcza marchew*. **Roots**: eaten by child shepherds, up until the 20^th ^century, preparation method unspecified, Mp [50].

### Cannabaceae

***Humulus lupulus *L. ON**: *chmiel zwyczajny*. **LN**: *chmiel*. **Inflorescences and fruits**: as a spice for honey, beer and bread dough, unspecified areas, at least until the 19^th ^century [27]. **Unspecified parts (probably shoots)**: as famine food, until the late 19^th ^century, Mp [48]. Nowadays used only in industrial breweries and in herbalism.

### Caprifoliaceae

***Sambucus nigra *L. ON/LN**: *bez czarny*, *dziki bez czarny*. **Fruits**: used to make wine and jam, all regions, still used [[31,34]:364]; in a few restricted areas a kind of soup (called *fafuła, borówka *or *gzica*) is made with the fruits, Zp, Lu, Op [[34]:364], Łd [[34]:364, [28]], Sw [28,65], Wp [28].

***Viburnum opulus *L. ON**: *kalina koralowa*. **LN**: *kalina*. **Fruits**: wine, Ps, Lu, Ds [[34]:367]; jam, Ps, Pk [[34]:367]; juice Pk, Sw, Lu [[34]:367]; used in some parts of SE Poland to make wine, juice and jam [31]. **NOTE**. More widely used for cough syrup, mainly in E Poland [[34]:367].

### Chenopodiaceae

***Atriplex *sp. pl. ON/LN**: *łoboda*. **LN**: *natyna*, *lebioda*. **Leaves**: boiled as an ingredient of potherb and soups, often mixed with *kasza *(cracked grain) or flour, widely used until the mid-20^th ^century, probably in most regions, no longer used [1,27,28], e.g. Lu [39,40], Kp [60], Pm [62]. **NOTE**. Probably under-recorded, usually not distinguished from *Chenopodium *(*Atriplex *and *Chenopodium *are not distinguished in the local folk taxonomy), probably the species used most commonly was ***A. patula *L**. although the only record referring to it is from Lu [39], where the leaves were cooked in a potherb called *wołoka*.

***Chenopodium album *L. ON**: *komosa biała*. ***LN***: *komosa*, *lebioda*, *łoboda*, *warmuz*. **Leaves**: boiled or fried with butter or lard, as a part of soup or potherb, often mixed with boiled potatoes or cracked cereals, mainly as poor people's and famine food, used even until the 1960s, in some rural areas, in all regions (except Ls) [[31,34]:363], e.g. Mp [42,46,48,50], Mz [37,52,53], Ps [28], Kp [28], Wp [57], Pm [62]; occasionally raw, chopped leaves added to other food used until the mid-20^th ^century, Sl, Mp, Pk, Lu, Łd, Mz, Ps, Wm, Pm [[34]:363]. Kurpie people (Mz) paid particular attention to rinsing leaves many times before using in cooking, they rinsed them until the water used for rinsing stopped being green [52]. **NOTE**. Although the above mentioned sources refer to *C. album*, probably other less common and not distinguished species were sometimes used together.

***Chenopodium bonus-henricus *L. ON**: *komosa strzałkowata*. **Leaves**: boiled, eaten alone, with potatoes or *kasza *(cracked cereals), sold in a vegetable market in Poznań, in 1953, Wp [28]. **NOTE**. Probably under-recorded.

### Convolvulaceae

***Convolvulus arvensis *L. ON**: *powój polny*. **LN**: *powój*. **Above-ground parts**: as famine food, until the turn of the 19^th ^and 20^th ^century, scalded and fried with lard, butter, cream, flour or eggs, Łd [54].

### Corylaceae

***Carpinus betulus *L. ON**: *grab pospolity*, *grab zwyczajny*. **LN**: *grab*. **Sap**: fresh, probably no longer used, Mz [53].

***Corylus avellana *L. ON**: *leszczyna pospolita*. **LN**: *orzech laskowy*, *leszczyna*, *laska*. **Fruit**: until recently widely collected from the wild (now only occasionally), eaten raw or in desserts, in all regions [27,31,35,46,53,54,61], often dried to be consumed at Christmas, e.g. Pm [62]. **Inflorescences**: female inflorescences grated to be included in bread, until the late 19^th ^century, Lu [39]; inflorescences eaten as famine food throughout Poland [27], dried, powdered and used to make bread called *obazina*, at least until the late 19^th ^century, Mp [46]. **NOTE**. Probably male inflorescences were used, the reference to female inflorescences may be a mistake, as they are much smaller than the male catkins.

### Cupressaceae

***Juniperus communis *L. ON**: *jałowiec pospolity*. **LN**: *jałowiec*. **Pseudo-fruits**: as a spice (commonly available in shops), especially in a sauerkraut stew called *bigos *and in *kiełbasa jałowcowa *sausages, probably in all regions; as meat preservative, Łd [65]; fermented into beer [27], often together with *Humulus lupulus *and honey/sugar, commonly until the mid-20^th ^century, most commonly in Mz [[31,52,34]:361], also in Wm, Ps, rarely in Zp, Kp [[34]:361] and Pm [[34]:361, [62]], today the tradition of juniper beer is fully alive only in the Kurpie region (Mz), where it is called *psiwo kozicowe *and has become a tourist attraction (Tomasz Madej, Warsaw University PhD student, researcher of Kurpie folk culture, personal communication); occasionally used to make wine, until the mid-20^th ^century [31], e.g. Op [59], Mp, Pk, Lu, Łd, Mz, Ps, Wm [[34]:361]; occasionally used to make liqueur, Lu, Mz, Ps, Pm, Wp [[34]:361], eaten raw by village children, in large quantities, at least until the mid-20^th ^century, Mz [52]. **NOTE**. From medieval times until at least the 18th century the "berries" constituted an important part of taxes paid by peasants to the landowners [57].

### Cyperaceae

***Scirpus sylvaticus *L. ON**: *sitowie leśne*. **Stem base**: the inner part of young shoots, as children's snack, raw, in spring, still occasionally used, Pk [36].

### Dennstaedtiaceae

***Pteridium aquilinum *(L.) Kuhn ON**: *orlica pospolita*. **Rhizomes**: preparation method not specified, as famine food, in the 19^th ^century, Mp [1].

### Empetraceae

***Empetrum nigrum *L. ON**: *bażyna czarna*. **LN**: *bażyna*. **Fruits**: eaten by "ordinary people", but little appreciated, supposedly causing headaches, until the 18th century, NE Poland [25].

### Equisetaceae

***Equisetum *sp. ON/LN**: *skrzyp*. **Strobils**: *szypułki*, as famine food in spring, raw, Mp [1,42,45], or cooked in soup, Mp [65], until the late 19^th ^century; the above mentioned references must pertain either to ***E. arvense *L**. or to ***E. telmateia *Ehrh**., which are both common in the Carpathians, and are the only horsetails to produce separate strobil-bearing shoots in spring. **Unspecified parts**: one informant from Wielkałąka stated that people used *Equisetum *to make potherb (*jarmuż*) in a similar fashion as with *Urtica *and *Chenopodium*, probably until the turn of the 19^th ^and 20^th ^century, Kp [61].

### Ericaceae

***Calluna vulgaris *(L.) Hull ON**: *wrzos pospolity*, *wrzos zwyczajny*. **LN**: *wrzos*. **Seeds**: used as an ingredient of famine bread, which was "astringent and dark", until the early 20^th ^century, Mz [53].

***Vaccinium myrtillus *L. ON**: *borówka czarna*. **LN**: *czarna jagoda*, *borówka*, *borowina*, *czernica*. **Fruits**: one of the most commonly collected wild foods, used in all regions [27,31,59], raw, in desserts and comfits, dried like raisins or used with milk and cream, throughout Poland [65]; juice made by covering berries with sugar, Sw [56]; dumpling filling, Pk [55]; as the main ingredient of fruit soups (combined with dairy products), Mp [45,48,49,63], Mz [53], Łd [54], Pk [55], Kp [61]; dried as a spice in winter, Kp [60].

***Vaccinium oxycoccos *L**.(syn. *Oxycoccus palustris *Pers.) **ON**: *żurawina błotna*. **LN**: *żurawina*, *klukwa*. **Fruits**: raw, in juices, boiled in sauces, made into desserts with flour, still used in at least one of these forms in most regions [27,31], e.g. Mz [53,35], Mp [50,35], Pm [35,62], Pk, Lu, Łd, Ps, Wm, Kp [35].

***Vaccinium uliginosum *L. ON**: *borówka bagienna*. **LN**: *pijanica*, *łochynia*, *wochynia*, *bagnówka*. **Fruits**: raw or in preserves, often used as an admixture to *V. myrtillus *dishes, its intoxicating effect, resembling that of alcohol, is widely known and reflected in folk names, attitudes towards edibility differ from village to village, widely collected in the 1960s, now used more rarely [35], all regions [[31,33]:310], e.g. Mz [53], Pm [62]; as the main ingredient of soup, Pm [62].

***Vaccinium vitis-idaea *L. ON/LN**: *borówka brusznica*, *borówka czerwona*. **LN**: *brusznica*, *borówka*. **Fruits**: raw, in juices or boiled, in preserves, jellies, sauces, used in at least one of these forms in all regions [31,65], e.g. Mp [35,48,50,63], Sl [35], Mz [35,53], Łd [54], Pk [55], Sw [56], Kp [35,59], Op [61], Lu, Ps, Wm [35], Pm [35,62].

### Euphorbiaceae

***Euphorbia peplus *L. ON**: *wilczomlecz ogrodowy*, *ostromlecz ogrodowy*. **Whole plant inlcuding roots**: boiled with milk for a famine soup, until the turn of the 19^th ^and 20^th ^century, Łd [54].

### Fabaceae

***Trifolium *sp. pl. ON/LN**:*koniczyna*. Probably mainly both ***Trifolium repens *L**. and ***T. pratense *L. Inflorescences**: preparation method not specified, as famine food, until the mid-20^th ^century, Sl [1], Mp [45], Mz, Ps, Wm [35].

***Vicia *sp. pl. ON/LN**: *wyka*. **Seeds**: sporadically ground and added to flour, to make bread, throughout the country, until the early or mid-20^th ^century [31], e.g. Op [59].

### Fagaceae

***Fagus sylvatica *L. ON**: *buk pospolity*, *buk zwyczajny*. **LN**: *buk*, *buczyna*, *bukiew*. **Fruits**: raw, as children's snack, or roasted in the stove, widely used until the beginning of the 20^th ^century, now rarely, Mp [[46,50,34]:359], Pk [[36,34]:359], Lu [85], Sl, Łd, Wm, Pm, Kp, Wp, Zp, Ds [[34]:359], Mz [53], Op [59], pressed to make oil, until the early 20^th ^century, Pk, Lu [[34]:359]; ground, as an addition to bread, until the early 20^th ^century, Lu [[31,33]:322, [34]:359], Pm [[34]:359]; roasted to make a drink, Pm [62]. **NOTE**. At least until the 19^th ^century sold in markets in the south [1] and south-east of the country [64]. **Leaf buds**: raw, probably until the turn of the 19^th ^and 20^th ^century, Mp [27].

**folk species 'DĄB' (= *Quercus *sp. pl.). ON/LN**: *dąb*. Both common species of oak, i.e. ***Quercus robur *L**. and ***Q. petraea *(Mattuschka) Liebl**. (syn. *Q. sessiliflora *Salisb., *Q. sessilis *Ehrh.) have been used indiscriminately. **Fruits**: ground and added to flour to make bread, as famine food until the mid-20^th ^century, Mp, Pk, Lu, Łd, Ps, Pm [[33]:322, [34]:359], Mz [[52,33]:322, [34]:359]; cracked (as *kasza*) and boiled, sometimes with milk, until the 20^th ^century, Mz [[34]:359, [52]]; roasted as coffee substitute, until the mid-20^th ^century, might be still used by single individuals, all regions except Ls and Op [[34]:359], e.g. Pm [62]. **Leaves**: as preservative in lacto-fermented cucumbers [30], probably most regions, e.g. Pk [55]; as a base for baking bread, partly to prevent the bread from sticking and partly to flavour the bread, mainly Lu, Ps, Wm, more rarely Mp, Pk, Kp, Ds, Ls, Zp [[32]:265].

### Grossulariaceae

***Ribes nigrum *L. ON/LN**: *porzeczka czarna*. **LN**: *smrodynia*. **Leaves**: leaves are widely used as a basic condiment for lacto-fermented cucumbers throughout Poland, nearly exclusively from garden grown bushes [30,65]. **Fruits**: preparation method not specified, collected from the wild until the mid-20^th ^century, Łd [54].

***Ribes *sp. ON/LN**: *porzeczka*. **Fruits**: eaten raw by shepherds, at least until the early 20^th ^century, Mp [63]. **NOTE**. This reference must pertain to either ***Ribes alpinum *L**. or ***Ribes petraeum *Wulfen **(syn. *R. carpaticum *Schult.), or both, as these are the only two *Ribes *species occurring in the Tatra Mts (excluding *R. grossularia *called a different folk name) [70].

### Lamiaceae

***Galeopsis *sp. ON**: *poziewnik*. **LN**: *dziomber, ziomber*. **Whole plant**: boiled as famine food, served with potatoes, oat flour, milk or butter, probably until the turn of the 19^th ^and 20^th ^century, Mp [45].

***Glechoma hederacea *L. ON**: *bluszczyk kurdybanek*. **LN**: *kurdybanek, kudron*. **Leaves**: as a spice for soups, until the beginning of the 20^th ^century, Pk [44], Mp [51], Op [59], Sl [62], unspecified part of S Poland [65].

***Melittis melissophyllum *L. ON**: *miodownik melisowaty (miodownik wielkokwiatowy*). **Leaves**: as famine food, preparation method unspecified, until the late 19^th ^century, Mp [1].

***Mentha arvensis *L. ON**: *mięta polna*. **Leaves**: as seasoning of *warmuz *soup composed of *Chenopodium album *and *Urtica dioica *leaves, used until the early or mid-20^th ^century, Mp [50].

***Mentha longifolia *(L.) Hudson**. (syn. *Mentha longifolia *(L.) L.) **ON**: *mięta długolistna*. **LN**: *końska mięta*. **Leaves**: seasoning for dumplings (*pierogi ruskie*) stuffed with fresh cheese, potatoes and fried onion, still occasionally used, Pk [36].

***Origanum vulgare *L. ON**: *lebiodka pospolita*. **Flowering tops**: beer condiment, in the 18th century [25]. **NOTE**. The Latin name of *O. vulgare *was used in two literature sources as a synonym of the Polish name of *Chenopodium *(i.e. *lebioda*) [31,44]. As the Polish name of *O. vulgare *is nearly identical (i.e. *lebiodka*), this must have been a mistake made by ethnographers looking up Latin names. In Poland used rather as medicine than spice [65], although the above mentioned name affinity with *Chenopodium *may indicate a forgotten culinary use.

***Thymus pulegioides *L. ON**: *macierzanka zwyczajna*. **LN**: *macierzanka*. **Leaves**: eaten together with *szczerboc *leaves (probably *Cirsium rivulare*), after scalding, used probably as a spice, until the turn of the 19^th ^and 20^th ^century, Mp [46]. **NOTE**. Recorded as *macierzanka *(i.e. *Thymus sp.*) but *T. pulegioides *is the only wild *Thymus *species from the described area.

***Thymus serpyllum *L. ON**: *macierzanka piaskowa*. **LN**: *macierzanka*. **Whole plants**: as a spice for dumpling (*pierogi*) fillings and soups, still used, Pk [55].

### Lemnaceae

***Lemna minor *L. ON**: *rzęsa drobna (rz. mniejsza*). **LN**: *rzęsa*. **Whole plants**: as famine food, blanched and fried with lard, butter, cream, flour or eggs, until the 20^th ^century, Mz [53].

### Liliaceae

***Allium ursinum *L. ON/LN**: *czosnek niedźwiedzi*. **LN**: *zajęczy czosnek*. **Bulbs**: as condiment, with meat dishes, recorded from the village of Łapsze Niżne, in the 1980s, Mp [49]. **Leaves**: raw, as a snack in small quantities, modern use initiated by the media; Pk (personal observation).

***Maianthemum bifolium *(L.) F.W. Schmidt **(syn. *Majanthemum bifolium *(L.) DC.).**ON**: *konwalijka dwulistna*. **LN**: *ptasie winko*. **Fruits**: collected by children for making wine, until the 20^th ^century, Mz [53].

### Malvaceae

***Malva neglecta *Wallr**. (syn. *Malva rotundifolia *L.) **ON**: *ślaz zaniedbany*. **LN**: *babi chleb*, *ślaz*. **Young leaves**: collected by "peasant women" for potherb, NE Poland, until the 18th century [25]. **Immature fruits**: raw, still eaten as children's snack, Sw (personal observation). **Seeds**: ground as an addition to bread, sporadically until the mid-20th century, Wp [[31,33]:322].

***Malva sylvestris *L. ON**: *ślaz dziki*. **LN**: *serek*, *babie serki*, *ślaz*. **Leaves**: according to Kluk [25], in the 18^th ^century, it could be used for potherb like *Malva neglecta*. **Immature fruits**: raw, eaten by children, Łd [54], Mz [53].

***Malva *sp. ON/LN**: *ślaz*. **Unspecified parts**: eaten until the 19^th ^or the beginning of the 20^th ^century, Wp [58]. **NOTE**. This reference may refer to both *M. neglecta *and *M. sylvetris*.

### Oxalidaceae

***Oxalis acetosella *L. ON**: *szczawik zajęczy*. **LN**: *zajęczy szczaw*, *zajęcza kapusta*. **Leaves**: raw, Mp [[45,46,50,34]:357], Łd [[54,34]:357], Mz [53], Pk, Sl, Sw, Op, Lu, Ps [[34]:357], Wp [58]; cooked in soups, Mp, Lu, Pm [[34]:357]. **NOTE**. Used widely until the 19^th^–20^th ^century, nowadays used only as a snack during woodland walks, but its edibility is popularly recognized by ordinary people.

### Pinaceae

***Abies alba *Mill. ON**: *jodła pospolita*. **LN**: *jodła*. **Young shoots**: covered with sugar to make syrup, mainly as a children's snack, probably still used, sold to tourists in the 1980s, Mp [49],

***Picea abies *(L.) H. Karst**. (syn. Picea excelsa (Lam.) Link.) **ON**: świerk pospolity. **LN**: świerk, smerek. **Young shoots**: raw, covered with sugar to make syrup, as children's snack, still used, Mp [49]; raw, as famine food, until the late 19^th ^century, Mp [42]. **Male inflorescences**: *májki*, raw, as a snack and famine food, until the late 19^th ^century, Mp [27,45]. **Young cones**: as famine food, until the late 19^th ^century, Mp [42].

***Pinus cembra *L. ON**: *sosna limba*. **LN**: *limba*. **Male inflorescences**: *májki*, raw, as a snack and famine food, until the late 19^th ^century, Mp [27,45]. **Seeds**: eaten as a snack at least until the 19^th ^century, Mp [45].

***Pinus sylvestris *L. ON**: *sosna zwyczajna*. **LN**: *sosna*. **Young shoots**: covered with sugar to make syrup, in May, still occasionally gathered, treated as a healthy snack protecting from colds, but included here because it is eaten by children in larger amounts for its taste, used probably in all regions, e.g. Pk [55], Sw [56]; **Young needles**: dried and ground, as famine food, to make bread together with rye, barley and pea flour, until the turn of the 19^th ^and 20^th ^century, Mz [52].

### Plantaginaceae

***Plantago lanceolata *L. ON**: *babka lancetowata*. **LN**: *babka*. **Leaves**: young leaves, boiled "like cabbage", until the turn of the 19^th ^and 20^th ^century, Mp [49].

### Poaceae

***Bromus secalinus *L. ON**: *stokłosa żytnia*, *stokłosa kostrzeba*. **LN**: *stokłosa*. **Seeds**: boiled to make gruel (as a kind of *kasza*) or ground and used as an ingredient of famine bread, until the mid-20^th ^century, Mp, Pk [[31,33]:322, [34]:358], Łd [54], Mz [53]; ground to make flatbread Mp, Pk, [[34]:358], preparation method not specified, Sw [[34]:358].

***Dactylis glomerata *L. ON**: *kupkówka pospolita*. **LN**: *kupkówka*, *rżniączka*. **Stem base**: the inner part of young shoots still occasionally eaten as a children's snack, Pk [36].

***Elymus repens *(L.) Gould **(syn. *Agropyron repens *(L.) P. Beauv.) **ON**: *perz właściwy*. **LN**: *perz*. **Rhizomes**: dried, powdered into flour, added to cereal flour to make dough bread or flatbread even until the mid-20^th ^century, mainly Mp, Pk, also Sl, Lu, Sw, Łd, Wm, Pm, Kp [[31,33]:322, [34]:356], Mz [[31,33]:322, [34]:356, [53]]; bread made of *Tilia *cambium and *E. repens *rhizomes was called *pachana*, Mp [46]; dried, powdered rhizomes cooked into gruel, Sl, Mp [[34]:356]; powdered rhizomes cooked in a soup, Sl, Pk, Łd, Kp [[34]:356]; might have been used to make beer until the 18th century [25].

***Festuca pratensis *L. ON**: *kostrzewa łąkowa*. **Fruits**: preparation method not specified, probably used until the end of the 19^th ^century or the first half of the 20^th ^century, Łd [53]. **NOTE**. It may have been used in a similar fashion as *Glyceria fluitans*.

***Glyceria fluitans *(L.) R. Br. ON**: *manna jadalna*. **LN**: *manna*. **Seeds**: ground used to make various forms of bread, until the early 20^th ^century, Mp [49], Lu [[34]:358], Mz [52]; cracked and boiled in a gruel, until the early 20^th ^century, Mp, Pk, Lu, Mz, Op, Ps, Wp, Wm [[34]:358]. **NOTE**. From medieval times until at least the 18^th ^century the seeds constituted an important part of taxes paid by peasants to the landowners [57]. An article of commerce until the 19^th ^century, even exported to Germany [1].

***Glyceria plicata *Fries**(syn. *Glyceria notata *Chevall.) **ON**: *manna fałdowana*. **LN**: *manna*. **Seeds**: formerly used in the same manner and together with *Glyceria fluitans*, until the early 20^th ^century, Mz [52]. **NOTE**. Probably under-recorded, used like *G. fluitans *and not distinguished from it.

### Polygonaceae

***Polygonum lapathifolium *L**. (syn. *P. nodosum *Pers.) **ON**: *rdest szczawiolistny*. **LN**: *rdest*. **Whole shoots**: famine food, scalded and fried with lard, butter, cream, flour or eggs, until the early 20^th ^century, Łd [54].

***Polygonum *sp. ON/LN**: *rdest*. **Leaves and seeds**: probably used for cooked potherb or soup, until the early 20^th ^century, Mp [49]. **NOTE**. Originally identified as *P. aegule *(*sic*!) (= *P. aviculare *ssp. *aequale*), but from the description of the place of collection ("shady places, forest edges and near water") we can assume that it incorporated several species from the genus, including *P. hydropiper *(L.) Spach., by far the commonest in such habitat in the Carpathians, and *P. lapathifolium *L.

***Rumex acetosa *L. ON**: *szczaw zwyczajny*. **LN**: *szczaw, scow, kwasielec, kapuśnica*. **Leaves**: raw as children's food and cooked used as an ingredient of soup (*zupa szczawiowa*), in all regions, other ingredients of this soup traditionally include flour, cream and either boiled egg, *kasza *or potatoes. Still occasionally collected from the wild, the last kind of green vegetable collected from the wild in all regions [27,31,45,46,53,54,60]. **Roots**: according to Kluk bread could be made out of the root in times of famine, in the 18th century [25]. **NOTE**. Usually not distinguished from *Rumex acetosella*, a smaller, very similar species, which tastes virtually the same, and is used in a similar fashion.

***Rumex acetosella *L. ON**: *szczaw polny*. **LN**: *szczaw*. **Leaves**: collected, eaten raw or cooked in soup, still used, Mp [48-50], Op [59], Pm [62]. **NOTE**. Probably under-recorded, usually not distinguished from *Rumex acetosa*, a larger, very similar species, which tastes virtually the same, and is used in a similar fashion.

***Rumex crispus *L. ON**: *szczaw kędzierzawy*. **Leaves**: fermented, mixed with flour, as famine food in the late 19^th ^century [1]. **Seeds**: could be made into flour and bread, in the 18th century [25].

### Polypodiaceae

***Polypodium vulgare *L. ON**: *paprotka zwyczajna*. **LN**: *słodyczka*. **Rhizomes**: raw, shepherd's snack, until the mid-20^th ^century, probably no longer used, Mz [52], Mp [50,63].

### Ranunculaceae

***Ranunculus ficaria *L**. (syn. *Ficaria verna *Huds.) **ON**: *ziarnopłon wiosenny, jaskier wiosenny*. **Leaves**: young, boiled or raw, garnished with *barszcz *(i.e. probably beetroot soup) and butter or lard, until the late 19^th ^century, Lu [40].

***Nigella *sp. ON/LN**: *czarnuszka*. **Seeds**: collected from the wild as spice on top of bread, Mz, [53]. **NOTE**. Reported as *Nigella sativa *L. which is only a rare escape from cultivation. It is more likely that the reference pertains to the commoner *N. arvensis *L. occurring as an arable weed in that area [67].

### Rosaceae

***Alchemilla *sp. ON/LN**: *przywrotnik*. **LN**: *gąsiorka*. **Leaves**: boiled, as famine potherb, until the late 19^th ^century, Mp [45].

**folk species 'GŁÓG' (= *Crataegus *sp. pl.) ON/LN**: *głóg*. The local folk taxonomy does not make any distinction and all the three common native species are collected, i.e. ***C. monogyna *Jacq**., ***C. laevigata *(Poir.) DC**. (syn. *C. oxyacantha *Auct. non L.) and ***Crataegus calycina *Peterm**.(including *C. rhipidophylla *Gand. = *C. curvisepala *Lindman) as well as their hybrids (personal observation). **Fruits**: in the 18th century "eaten by simple people" [25], nowadays fruit mainly made into wine, sporadically jams, or eaten as a children's snack, most regions, e.g. Sl [35,47], Pk [55], Mp, Lu, Sw, Mz, [35], Kp [35,61].

***Fragaria vesca *L. ON**: *poziomka pospolita*. **LN**: *poziomka*, *czerwona jagoda*. **Fruits**: commonly collected and widely used, mainly raw, sometimes in desserts and comfits, all regions [27,31,35,54,59,60,62,63].

***Malus domestica *Borkh. ON**: *jabłoń domowa*. **LN (for wild forms)**: *płonka*. **Fruits**: wild and semi-wild apples collected, especially in the past when there were fewer orchards, eaten raw, dried, cooked into compotes or commonly included in sauerkraut, in most regions, commonly until the mid-20^th ^century, now rarely, e.g. Sl [47], Mp [46], Mz [52,53], Wp [57]. **NOTE**. The large majority of seemingly wild apples are *M. domestica*, which escaped from cultivation, the native *Malus sylvestris *Miller is extremely rare (Prof. Jerzy Zieliński, Insitute of Dendrology, Polish Academy of Sciences, spoken communication), so the references to eating "wild" apples pertain to the former species.

***Potentilla anserina ***L. **ON**: *pięciornik gęsi*. **Young shoots**: raw, as salad, in the mid-20^th ^century, Ps [65].

***Prunus avium *L**. (syn. *Cerasus avium *(L.) Moench) **ON**: *czereśnia ptasia*. **LN**: *czereśnia*, *cześnia*, *trześnia*. **Fruits**: raw, eaten mainly by children, the edibility of wild cherries, whose range is restricted to southern Poland only, is widely known, use under-recorded, Sl [47], Pk (personal observation). **Solidified sap**: as a children's snack, still used, Mp [46], Pk (personal observation).

***Prunus padus *L**.(syn. *Padus avium *Mill.) **ON**: *czeremcha ptasia*. **LN**: *korcipa*, *czeremcha*. **Fruits**: raw, eaten by children, no longer used, Sl [47], unspecified areas [27,65]; wine, until the mid-20^th ^century, no data on current use, Op [59].

***Prunus spinosa *L. ON**: *śliwa tarnina*. **LN**: *tarnina*, *tarka*, *ciarka*. **Fruits**: wine, juice (mainly obtained by boiling with water), compotes, jams, raw (as children's snack), all regions [[31,34]:366], e.g. Mz [53], Op [59], Kp [61], Lu [31]. **NOTE**. The use of *P. spinosa *for making jams is restricted mainly to Kraśnik area (Lu), where it has been recorded from many localities, other types of use are spread more evenly around the country, however most common in S and SE Poland [[31,34]:366].

**folk species 'GRUSZA' (= *Pyrus *sp. pl.) ON/LN**: *grusza*. **LN**: *ulęgałka*, *dzika grusza*. Both feral specimens of the domestic ***Pyrus pyraster *(L.) Burgsd**. and the wild ***Pyrus communis *L. em. Gaertner **are commonly found in forest and field margins and their fruits have been collected indiscriminately. **Fruits**: raw, dried to be used later in compotes, boiled and pasteurized or pickled in vinegar, in most regions, widely used until recently, e.g. Ps [38], Pk [43], Mp [46], Mz [52,53], Łd [54].

**folk species 'DZIKA RÓŻA' (= *Rosa *sect. *Caninae*) ON/LN**: *róża*. **LN**: *dzika róża*. **Fruits**: wine and preserves, as well as a medicinal plant [55,65] and occasionally as children's snack [27], some of the present uses may be the result of press articles and herbalist guides [49], probably in most regions, e.g. Pk [55], Mp [35,49], Sw, Lu, Sl, Mz, Ps, Kp, Pm [35]; as baby food, ground in a handmill, cooked with milk, until the turn of the 19^th ^and 20^th ^century, Mp [45]. **NOTE**. All the cited ethnobotanical works report the use of ***Rosa canina *L., **the commonest species out of several species of this section found in Poland. However, other rarer species from the section *Caninae *are probably collected along with *R. canina *and under-recorded.

***Rubus idaeus *L. ON**: *malina właściwa*. **LN**: *malina*. **Fruits**: raw, juice, syrups, wines and desserts, one of the most commonly collected wild plants, still used, in all regions [27,31,65].

**folk species 'JEŻYNA' (= *Rubus *L. subgenus *Rubus*). ON/LN**: *jeżyna*. **LN**: *ostrężyny*, *czernice*, *dziady*, *drapaki*. One of the most commonly collected wild fruits [1,27,59]. **Fruits**: raw, juice, collected in all regions, more often in the south, where they are more abundant [[31,34]:362, [65]]; jam, wine, all regions, except Ps [[34]:362]; cooked as the main ingredient of a flour-thickened soup, Mz [53]. **NOTE**. The taxonomy of *Rubus *is very complicated due to the existence of numerous agamic pseudo-species [84]. Hence ethnographic literature is abundant with erroneous Latin names given to blackberries. In Bohdanowicz's review [31] as well as Jędrusik's thesis [35] all blackberries were misnamed as ***Rubus caesius *L**., the species which in fact has the least tasty berries and is collected only occassionally, in one study [47] local people emphasized that they collect all blackberry species except for *R. caesius*! So the species most often collected for consumption are rather species from the section Rubus than the sour, small-fruited *R. caesius*. Obviously the most commonly collected species are those which are most frequent and have the largest distribution ranges. At present 80 *Rubus *species from section Rubus have been recorded in Poland [84], by far the commonest are ***R. plicatus *Weihe et Nees, *R. nessensis *Hall **(syn. *R. suberectus *Weihe), and ***R. hirtus *Waldst. & Kit., **and from the disproportion in the number of their localities and the localities of other species [84], we can estimate that at least half of all the collected blackberries is made up of these three species. The last of these species, *R. hirtus*, is one of the commonest plants in the Carpathian beechwoods and firwoods, where it is collected in large quantities (personal observation).

***Rubus saxatilis *L. ON**: *malina kamionka*. **LN**: *kamionka*. **Fruits**: raw or made into juice, until the mid-20^th ^century, probably still occasionally collected, Mz [53], Łd [54].

***Sorbus aucuparia *L. emend. Hedl. ON**: *jarząb pospolity*, *jarząb zwyczajny*. **LN**: *jarzębina*, *jarząb*. **Fruits**: wine, sporadically, but in all regions [[31,34]:367]; liqueur, Wm, Ps, Mz, Lu, Mp, Wp, Op, Ds [[34]:367]; jam, Sl, Wm, Ps, Mz, Wp, Ds, Mp [[34]:367]; juice, Wp, Sw, Mz, Lu, Mp, Pk, Wm [[34]:367]; dried for further use, until the early 20^th ^century, Mz [52]. **NOTE**. Commercially produced liqueur, called *jarzębiak*, made with its fruits has been commercially available in shops for decades.

### Tiliaceae

**folk species 'LIPA' (= *Tilia *sp. pl.) ON/LN**: *lipa*. The two native species from this genus, the commoner ***Tilia cordata *Miller **and the less common, restricted to the south of the country, ***Tilia platyphyllos *Scop**. are not distinguished by ordinary people and were used in the same fashion. **Leaves**: boiled together with the leaves of vegetables, as famine food, in 1885, Wm [27]; chopped to be included in bread, as famine food, in the 19^th ^century, Lu [39]. **Cambium**: dried and powdered into flour, during spring famines, bread made with *Tilia *and *Elymus repens *rhizomes flour was called *pachana*, until the turn of the 19^th ^and 20^th ^century, Mp [46]. **Opening leaf buds**: fermented in wooden containers, later used to make soup, until the early 20^th ^century, Mz [52]. **Sap**: one informant from the village of Rzepnik states that he has occasionally drunk the sap of both species in spring, following a local tradition, Pk [36]. **Inflorescences**: the whole inflorescences of both *Tilia *species are commonly collected in June-July, dried and used for making infusions, throughout Poland [25,35,36,53-56,65], this infusion is believed to have medicinal properties, e.g. against colds etc. but up to the 20^th ^century used to be drunk on an everyday basis in colder months. **NOTE**. Kluk, in the 18^th ^century, described his experiments with making chocolate out of ground flowers and fruits and praised the oil extracted from fruits, but his activities were probably influenced by literature, in the 18th century [25].

### Trapaceae

***Trapa natans *L. ON/LN**: *kotewka orzech wodny*. **LN**: *kotwiczka*. **Fruits**: eaten after scalding, which helped to open the nut, until the mid-20^th ^century, Op [59]; unspecified preparation method, 19^th ^century, Lu [40]; in the past collected to be sold as a snack by Jewish merchants from Sandomierz, known there as *żydowskie orzechy *(i.e. *Jewish nuts*), Sw, Pk [85]. **NOTE**. Moszyński [27] stated that *Trapa *use by Poles died out (partly due to the near-extinction of the species), in contrast to the southern and eastern Slavs who still used it.

Urticaceae **folk species 'POKRZYWA' (= *Urtica *sp. pl). ON/LN**: *pokrzywa*. **LN**: *żegawka*, *zagawka*, *żagawka*. The two native species of this genus, ***Urtica dioica *L**. and ***U. urens *L**. are usually treated as one folk species and were used in the same way, sometimes together e.g. [53,54]. **Tops**: collected in spring and early summer, often as famine food, more rarely as normal food, scalded, boiled or/and fried, eaten with potatoes, *kasza*, eggs or fat, often mixed with other wild green vegetables, in all regions, until the mid-20^th ^century [1,31], as *Urtica *sp.: Lu [40], Mz [52], Kp [61], Mp [45,49], Pm [62], as *U. dioica*: Mz [53], Łd [54], Op [59], Mp [50], as *U. urens*: Mz [53], Łd [54]; chopped leaves of both species, with eggs and pepper were used as crayfish stuffing, until the early or mid-20^th ^century, Łd [54]. **NOTE**. After a few decades of neglect, nowadays tea or soup made with *Urtica dioica *leaves are consumed as an increasingly fashionable health food, and a part of urban culture.
